# Digital Gene Expression Analysis of Corky Split Vein Caused by Boron Deficiency in ‘Newhall’ Navel Orange (*Citrus sinensis* Osbeck) for Selecting Differentially Expressed Genes Related to Vascular Hypertrophy

**DOI:** 10.1371/journal.pone.0065737

**Published:** 2013-06-05

**Authors:** Cheng-Quan Yang, Yong-Zhong Liu, Ji-Cui An, Shuang Li, Long-Fei Jin, Gao-Feng Zhou, Qing-Jiang Wei, Hui-Qing Yan, Nan-Nan Wang, Li-Na Fu, Xiao Liu, Xiao-Mei Hu, Ting-Shuai Yan, Shu-Ang Peng

**Affiliations:** College of Horticulture and Forestry Sciences, Huazhong Agricultural University, Key Laboratory of Horticultural Plant Biology, Ministry of Education, Wuhan, China; Wuhan University, China

## Abstract

Corky split vein caused by boron (B) deficiency in ‘Newhall’ Navel Orange was studied in the present research. The boron-deficient citrus exhibited a symptom of corky split vein in mature leaves. Morphologic and anatomical surveys at four representative phases of corky split veins showed that the symptom was the result of vascular hypertrophy. Digital gene expression (DGE) analysis was performed based on the Illumina HiSeq™ 2000 platform, which was applied to analyze the gene expression profilings of corky split veins at four morphologic phases. Over 5.3 million clean reads per library were successfully mapped to the reference database and more than 22897 mapped genes per library were simultaneously obtained. Analysis of the differentially expressed genes (DEGs) revealed that the expressions of genes associated with cytokinin signal transduction, cell division, vascular development, lignin biosynthesis and photosynthesis in corky split veins were all affected. The expressions of *WOL* and *ARR12* involved in the cytokinin signal transduction pathway were up-regulated at 1^st^ phase of corky split vein development. Furthermore, the expressions of some cell cycle genes, *CYCs* and *CDKB*, and vascular development genes, *WOX4* and *VND7*, were up-regulated at the following 2^nd^ and 3^rd^ phases. These findings indicated that the cytokinin signal transduction pathway may play a role in initiating symptom observed in our study.

## Introduction

B is essential for higher plants [1], and soluble B content in soil and irrigation water is a crucial for determining the yield of agricultural products [2]. B deficiency (BD) has been reported in 132 plants in more than 80 countries [3]. It is well known that B deficiency will lead to different phenotypes in vascular plants, for example, the male sterility in wheat and rice [4], [5], the reduction of root biomass and nodules in legume [6], [7], the deformation of young leaves and the hypertrophy of petioles in castor bean [8], and the cracked bend veins of leaves in mulberry [9].

Citrus is an agriculturally and economically important fruit tree in the world. It is very sensitive to low B content in the soil. The occurrence of B deficiency has been reported in the major citrus producing countries, such as Spain, America, Brazil and China [3]. In China, large south and east areas contain extremely low level of soluble B (<0.25 mg kg^−1^) [10], [11]. Being a predominant region of naval orange production, Ganzhou an area of Jiangxi province in south China plays an important role in the country's citrus production. However, corky split veins of leaves usually occurred in main local cultivars, ‘Newhall’ navel orange (*Citrus sinensis* Osb.) [12], which declines the vigour of tree rapidly after the fruit set, and eventually affects fruit yield and quality in the coming years [13].

The primary function of B in higher plants is to form borate esters with apiose residues of rhamnogalacturonan II (RG-II) [14]. The formation of the complex is essential for cell wall structure and function [15] since it contributes significantly to the control of cell wall porosity [16] and tensile strength [17]. However, this participation does not seem to explain physiological phenomena of B deficiency documented in many other studies, for example, B deficiency increases the membrane permeability [18], [19] and the activity of proton-pumping ATPase in sunflower [20], causes an accumulation of phenolics through the stimulation of the enzyme phenylalanine-ammonium lyase (PAL) in tobacco and squash [21], [22] and alters amino acid profiles in white lupin [23]. Molecular genetic research have identified two types of B transporters: NIPs (nodulin-26-like intrinsic proteins), boric acid channels, and BORs, B exporters, which are both important for the uptake of B by roots, xylem loading and B distribution among leaves under B deficiency condition [2], [24], [25], [26], [27], [28], [29], [30], [31], [32]. In addition, other B-related proteins, for example, WRKY6, a low-B-induced transcription factor, is reported to be essential for Arabidopsis normal root growth under low-B condition [33], and the pathogenesis-related (PR) proteins from the PR-10 family are reported to be highly induced in low-B nodules during the legume-rhizobia interaction [34]. Recently, a quantitative trait locus (QTL) analysis for seed yield and yield-related traits under low and normal B conditions have been carried out in *Brassica napus* [35]. Despite the increasing studies on B deficiency in plants, little have been documented on corky split vein and the effect of the stress on leaf vein.

The purpose of this study was to get insight into the molecular mechanisms of corky split vein of ‘Newhall’ navel orange which was sandy cultured in a greenhouse under the ccondition of B deficiency. A genome-wide analysis of gene expression profiling at four phases during a long-term B deficiency treatment was performed in the experiment. Our results yielded numbers of DEGs related to the formation of corky split veins.

## Materials and Methods

### Plant materials and B treatments

‘Newhall’ navel orange (*C. sinensis* Osb.), grafted on Trifoliate orange (*Poncirus trifoliata* (L.) Raf.) was used in the experiment. The virus-free plants were obtained from the National Indoor Conservation Center of Virus-free Germplasms of Fruit Crops at Huazhong Agricultural University in Wuhan. According to the published method [36], [37], [38], the one- year plants grown in a greenhouse were washed with deionized water to remove surface contaminants, then were transplanted to the black pots containing B-free medium composed of quartz sand: perlite (1∶1, v/v). The plants were irrigated every other day with a modified Hoagland's No.2 nutrient solution containing either 0 (B deficiency, BD) or 0.25 mg l^−1^ (control, CK) B [38], [39]. The treatments started at the beginning of April, 2010 and terminated in May, 2011 when the visible symptom (corky split vein) of B deficiency appeared. Based on the morphological character of corky split vein development, both CK and BD veins collected at 28^th^ March, 2011 (CK1 and BD1), 7^th^ April, 2011 (CK2 and BD2), 16^th^ April, 2011 (CK3 and BD3) and 2^nd^ May, 2011 (CK4 and BD4) were used for the anatomical observation, DGE analysis, and quantitative real-time PCR (qRT-PCR). In addition, leaves collected on 2^nd^ May were analyzed for the total B concentration.

### Total B concentration measurement

Total B concentration was measured by the method of previous research [38]. 0.50 g of each sample was dry-ashed in a muffle furnace at 500°C for 6 h, followed by dissolution in 0.1 N HCl, and B was determined by ICP-AES.

### Light microscopy for cross-section observation of veins

Microscopy observation was performed on fresh lateral veins (LVs) according the method previously described [40]. The 3^rd^ LV with the lenghth of 0.5 centmeter (cm) near the petiole were collected (Figure S1, asterisked). LVs were immerged in the solution of formalin-acetic acid-alcohol (FAA) and vacuumized for 30 min. After fixation, the samples were routinely dehydrated in an ethanol series, and in the end they were transparentized in dimethylbenzene solution, embedded in paraffin, and sectioned at 6 µm by using microtome (Lecia RM-2255, Germany). The sections were transferred onto Superfrost Plus slides and dried for five days at 40°C–45°C, and then dyed by safranine-fast green.

### Digital gene expression profiling

The B treatment was terminated when the visible symptom appeared as described above. Leaf samples of four phases covering the whole gradient of variation in morphology and anatomy of corky split veins were collected for the following RNA isolation. The total RNA were isolated from veins (cutted as Figure S1, whole) of the four stages, and were named as libraries BD1, CK1, BD2, CK2, BD3, CK3, BD4, CK4 respectively. RNA extraction was performed according to the manufacturer's instructions of TRIzol reagent (TaKaRa, Dalian, China).

Quality and quantity analysis of total RNA, library construction, and sequencing were carried out at Huada Genomics Co., Ltd., Shenzhen, China. After the total RNA was extracted from the samples, mRNA was enriched by using the oligo (dT) magnetic beads. By adding the fragmentation buffer, the mRNA was interrupted to short fragments (about 200 bp), and then the first strand cDNA was synthesized by random hexamer-primer using the mRNA fragments as templates. Buffer, dNTPs, RNase H and DNA polymerase I were added to synthesize the second strand. The double strands cDNA was purified with QiaQuick PCR extraction kit and washed with EB buffer for ending repair and the addition of single nucleotide A (adenine). Finally sequencing adaptors were ligated to the fragments. The fragments were purified by agrose gel electrophoresis and enriched by PCR amplification. The library products were ready for sequencing analysis via Illumina HiSeq™ 2000. Then the raw reads data available as fasta files were deposited in the NCBI Sequence Read Archive (SRA) database (http://www.ncbi.nlm.nih.gov/Traces/sra_sub/sub.cgi?view=submissions) under eight corresponding run accession numbers, SRR824732 (CK1), SRR824755 (CK2), SRR825214 (CK3), SRR825191 (CK4), SRR825192 (BD1), SRR825193 (BD2), SRR825194 (BD3), and SRR825195 (BD4).

To get the clean reads, the dirty raw reads were removed from raw data before data analysis by filtering reads with adaptors, reads in which unknown bases were more than 10%, and low quality reads in which the percentage of the low quality bases of quality value ≤5 were more than 50%. Subsequently, the proportion of clean reads in raw reads of the eight libraries was classified.

The clean reads of eight libraries were mapped to reference sequences [41], [42], [43] (http://www.phytozome.net/search.php?method=Org_Csinensis) using SOAPaligner/soap2 [44] and the NCBI database (http://www.ncbi.nlm.nih.gov/). Mismatches no more than 2 bases were allowed in the alignment. The reads mapped to reference sequences from multiple genes were filtered. Subsequently, sequencing saturation analysis and the randomness assessments were carried out.

### Identification of differentially expressed genes

The gene expression level was calculated by using RPKM [45] method (Reads Per kb per Million reads), and the formula is shown as follows:
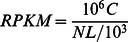
C represents the number of reads that uniquely aligned to a gene, N represents the total number of reads that uniquely aligned to all genes, and L represents the number of bases on a gene.

All libraries of clean reads were normalized to RPKM value to obtain normalized gene expression level. A strict algorithm [46] was performed to identify DEGs between two samples. Relative change threshold in pairwise comparisons across four phases was performed the absolute value of log_2_(BD-RPKM/CK-RPKM). The threshold with a FDR (False Discovery Rate) ≤0.001 and the absolute value of log2 Ratio ≥1 was used to judge the significance of gene expression difference.

For Gene Ontology (GO) enrichment analysis and pathway enrichment analysis, all DEGs were mapped to GO terms in the database (http://www.geneontology.org/) and pathway terms in KEGG database (http://www.genome.jp/kegg/), respectively.

### Quantitative real-time PCR analysis

To verify the DGE results, qRT-PCR was used for the investigation of DEGs expression levels. The qRT-PCR analysis protocol was performed as previously described [28]. The experiment was conducted with three replicates for each sample. Citrus actin gene was used as a normalizer and the relative expression levels of genes were presented by 2^−ΔΔCT^ (gene/actin). Primers of actin and DEGs for qRT-PCR were shown in Table S1.

## Results

### B deficiency caused leaf vein tissue hypertrophy

**Figure 1:**
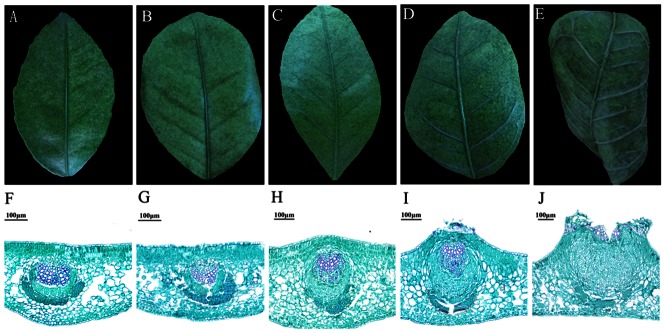
Defining developmental and anatomical characters in mature leaves. [A–E] Morphological performance of CK leaf [A], BD1 leaf [B], BD2 leaf [C], BD3 leaf [D], and BD4 leaf [E]. [F–J] Light micrograph of lateral vein of CK [F], BD1 [G], BD2 [H], BD3 [I], and BD4 [J].

**Figure 2:**
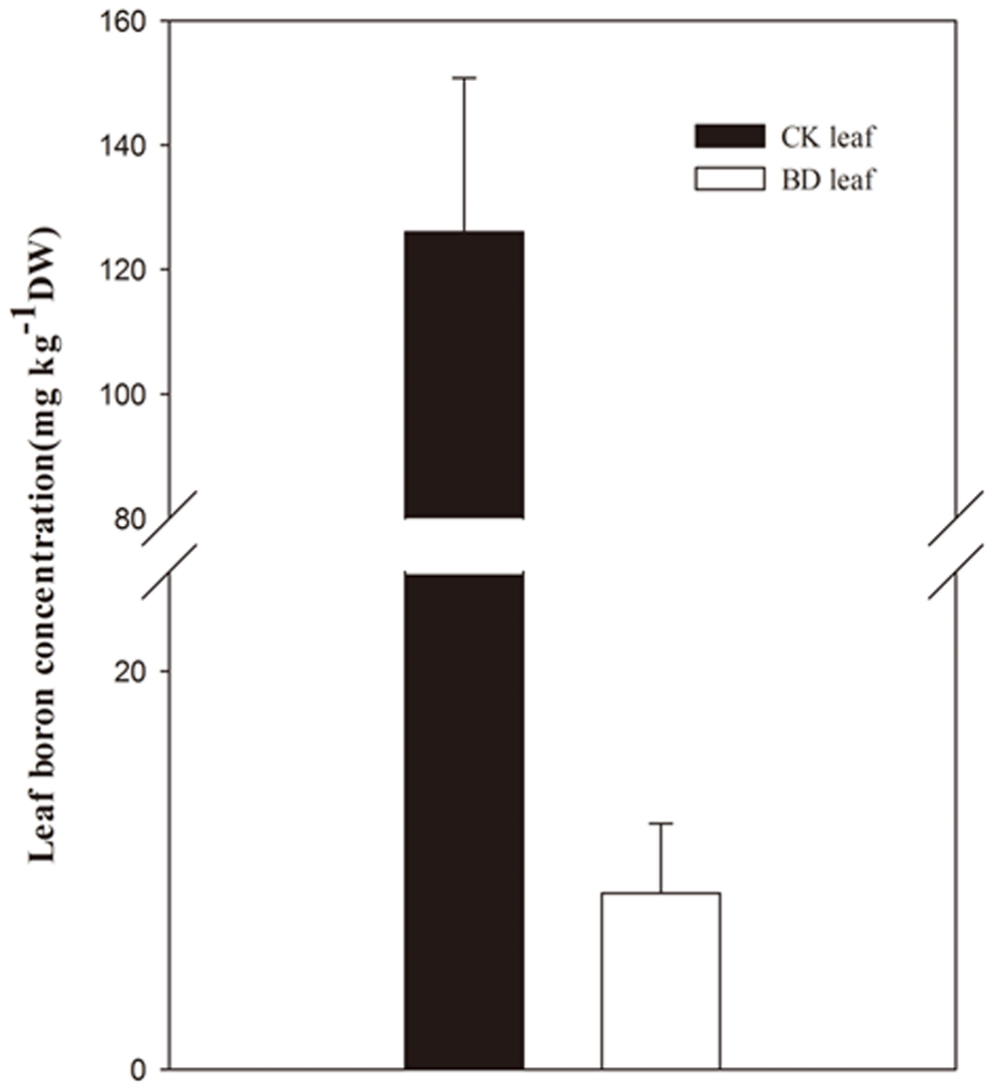
B concentration of whole leaf. Columns and bars represented the means and standard error of B concentration respectively (y-axis). Three replicates were used for leaf B concentration measure. DW, dry weight.

Corky split veins were observed in mature leaves of the B-deficient plants (Figure 1D, E) at approximately one year after treatments, and the result of determination on B concentrations of leaves showed that B level was extremely lower in BD treatment (8.86 mg kg^−1^DW) than that in CK treatment (125.98 mg kg^−1^DW) (Figure 2).

Morphological (Figure 1A–E) and anatomical (Figure 1F–J) characters were performed at the four stages in corky split leaf vein development to further create inventories of gene expression. As no obvious difference in morphology and anatomy among the CK veins of four stages (CK1–CK4) was observed (data not shown), thus, only one morphological or anatomical photograph was chosen to be shown as CK (Figure 1A, F). Correspondingly, the BD veins of the four stages which covered the whole gradient of variation in morphology and anatomy of corky split veins were selected for the following transcriptomic analysis. These stages included a phase (BD1, Figure 1B) of being similar with CK (Figure 1A), a phase (BD2, Figure 1C) of veins protruding from the blade, a phase (BD3, Figure 1D) of slight corking veins and a phase (BD4, Figure 1E) of seriously corky split veins.

Light micrographs of lateral veins exhibited a continuous variation of hypertrophy in vascular tissue compared with CK (Figure 1F–J). At the BD3 phase, the epidermis was destroyed by the proliferating vascular cylinder for the first time in terms of anatomical observation (Figure 1I).

### Analysis of DGE libraries and reads mapping

Being operated in ice, veins were separated from leaves (Figure S1, whole). Illumina HiSeq™ 2000 platform was used to perform high throughput sequences analysis on the eight citrus leaf vein libraries to investigate the transcriptomic response to B deficiency in the vein. More than 7.1 million total reads per library were obtained and above 99.21% of total reads were identified as clean reads (Figure S2) before mapping them to the reference database (Table 1).

**Table 1:** Major characteristics of eight libraries.

Summary		CK1	BD1	CK2	BD2	CK3	BD3	CK4	BD4
**Raw reads**	TN	7179660	7623813	7259087	7178343	7607537	7596798	7579221	7143559
**Clean reads**	TN	7132497	7565451	7209850	7132317	7554064	7536975	7528213	7091768
	TP	99.34%	99.23%	99.32%	99.36%	99.30%	99.21%	99.33%	99.27%
**Mapped reads**	TN	5415328	5834339	5488209	5488088	5720491	5618233	5609421	5356143
	TP	75.92%	77.12%	76.12%	76.95%	75.73%	74.54%	74.51%	75.53%
**Mapped genes**	TN	23290	22897	23433	23054	22374	23792	23422	23228
	TP	50.47%	49.62%	50.78%	49.96%	48.48%	51.56%	50.76%	50.33%
**Perfect match**	TN	4079188	4393468	4123087	4110504	4250953	4184929	4198715	4011645
	TP	57.19%	58.07%	57.19%	57.63%	56.27%	55.53%	55.77%	56.57%
**≤2 bp mismatch**	TN	1336140	1440871	1365122	1377584	1469538	1433304	1410706	1344498
	TP	18.73%	19.05%	18.93%	19.31%	19.45%	19.02%	18.74%	18.96%
**Unique match**	TN	2076283	2235674	2090868	2109145	2152356	2348318	2187769	2261917
	TP	29.11%	29.55%	29.00%	29.57%	28.49%	31.16%	29.06%	31.89%

TN, Total Number. TP, Total Percentage.

The total clean reads of eight libraries were mapped to a reference gene database of *C. sinensis*, in which 46147 unigenes were included. The randomness assessments showed that the reads in every position of reference gene distributed evenly and demonstrated highly similar tendencies in the eight libraries (Figure S3) which indicated that the randomness of RNA fragmentation was sufficient for subsequent bioinformatics analysis of gene expression. Over 5.3 million clean reads per library were successfully mapped to the reference database, and more than 22897 mapped genes per library were simultaneously obtained. In addition, 55.53%–58.07% of the clean reads were mapped to the unigene database perfectly, 18.73%–19.45% of the clean reads were mapped to the unigene database with ≤2 bp mismatch and 28.49%–31.89% of the clean reads were mapped to the unigene database with a unique match (Table 1).

To estimate whether the sequencing depth was sufficient for the transcriptome coverage, the sequencing saturation was analyzed in the eight libraries (Figure S4). With the number of reads increasing, the number of detected genes was increasing in the eight libraries. However, when the number of reads reached 3 million reads or higher, the growth rate of detected genes became flatten, which showed that the number of detected genes tended to be saturated.

### Changes in global gene expression in B-deficient leaf veins

Global gene expression of each sample of the four B-deficient phases (BD1–BD4) was assayed using the corresponding control phase (CK1/CK2/CK3/CK4) as a common reference. The rigorous algorithm method and the relative change threshold described above were applied to judge the significant difference of gene expression. The results showed that 7202 genes had the threshold in at least one of the pairwise comparisons, which were declared to be differentially expressed during the B deficiency course (Table 2). 1387 genes were differentially expressed between the BD1 and CK1 veins. Among these genes, 683 (49.24%) were up-regulated and 704 (50.76%) were down-regulated in response to B deficiency. Most of genes were up-regulated at the succedent phases, especially at the BD3 stage. 1986 (66.89%), 1920 (62.95), and 1662 (60.57%) genes were up-regulated at the BD3 phase compared with the CK3, BD1, and BD2 phase, respectively (Table 2).

**Table 2:** Differentially expressed genes across all libraries.

	CK1 vs. BD1	CK2 vs. BD2	CK3 vs. BD3	CK4 vs. BD4	BD1 vs. BD2	BD1 vs. BD3	BD1 vs. BD4	BD2 vs. BD3	BD2 vs. BD4	BD3 vs. BD4
**Total**	1387	418	2969	2168	798	3050	2350	2744	2150	710
**Up-regulated**	683	173	1986	1195	348	1920	1367	1662	1232	295
**Down-regulated**	704	245	983	973	450	1130	983	1082	918	415

All the genes mapped to the reference sequence were examined for their expression differences across the eight libraries. CK1, CK2, CK3, CK4, BD1, BD2, BD3 and BD4 represent eight libraries, respectively. Numbers of differentially expressed genes represent across sense transcripts, using the threshold with a FDR (False Discovery Rate) ≤0.001 and the absolute value of log2 Ratio ≥1 for controlling false discovery rates.

**Figure 3:**
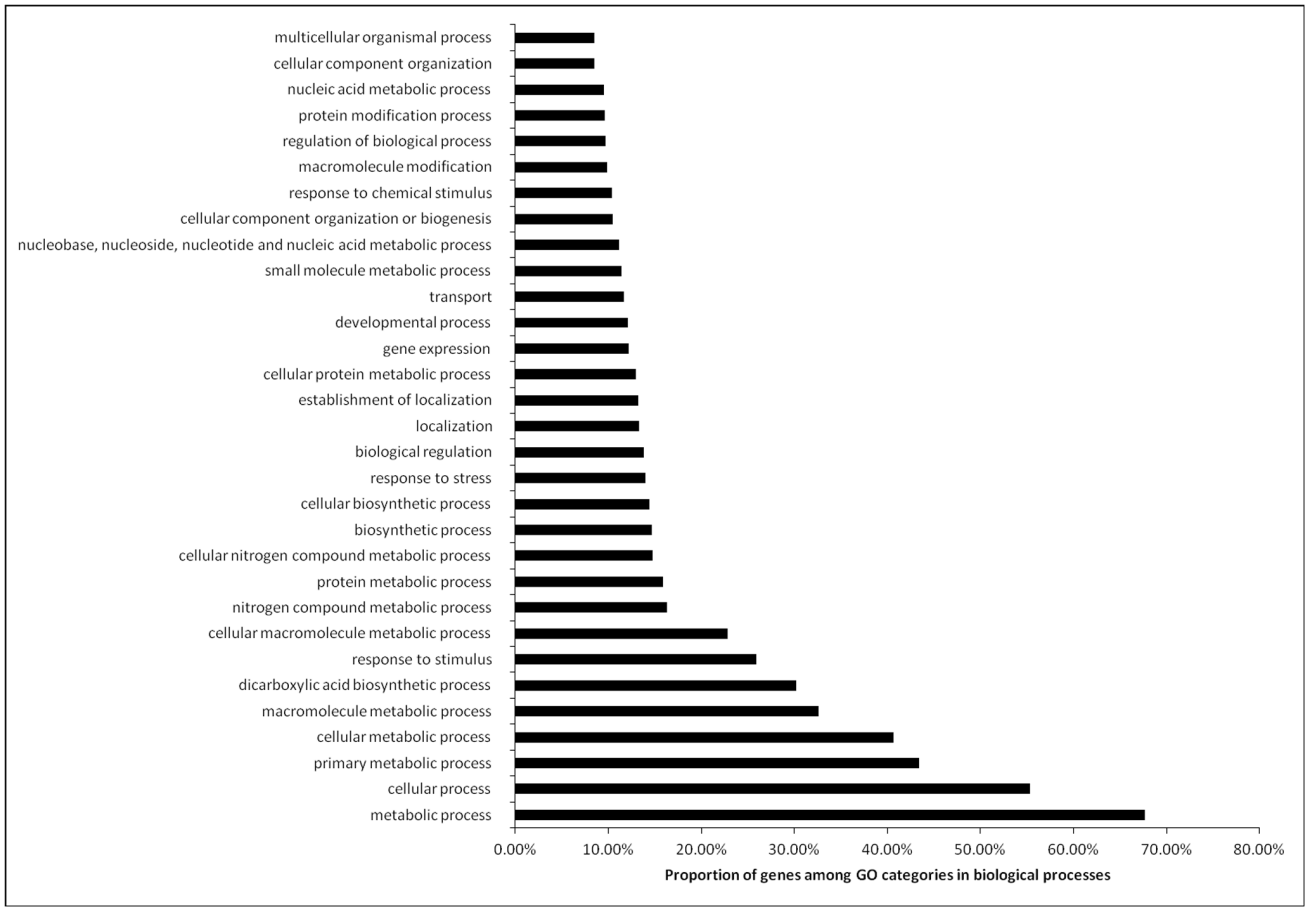
Gene classification based on gene ontology (GO) for differentially expressed genes in BD3 and CK3 libraries of citrus vein. Only the biological processes were used for GO analysis. The y-axis and x-axis indicated the names of clusters and the ratio of each cluster, respectively.

**Figure 4:**
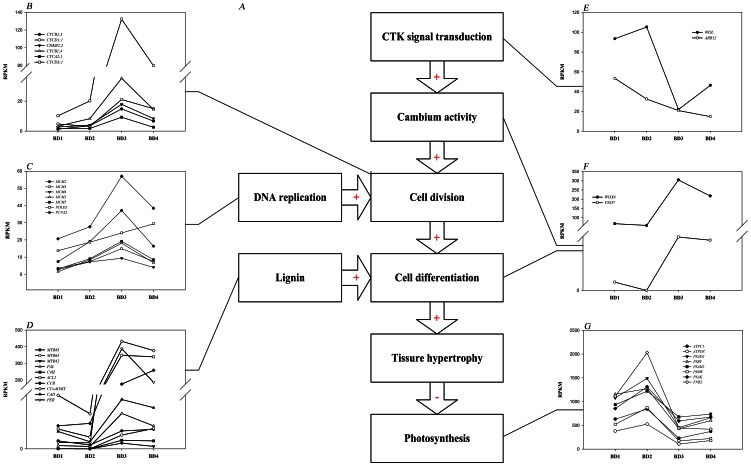
Overview of major pathways and their expression profiles during the corky split vein development. [A] Overview of major pathways in corky split vein caused by B deficiency. [B–G] Expression profiles of the DEGs involved in cell cycle [B], DNA replication [C] lignin biosynthesis [D], cytokinin signal transduction [E], vascular development [F], and photosynthesis [G] pathways from BD1 phase to BD4 phase. Pathways with transcripts that were up-regulated or down-regulated were indicated with + or −. CTK, cytokinin. DEGs, differentially expressed genes.

DEGs spanned over a wide variety of processes (pairwise comparison of BD3 and CK3 for example, Figure 3) and in functional groups (Table 3). All genes with altered expression in citrus vein at the four phases were mapped to GO terms (p-value≤0.05) in the database and were classified into several categories. The DEGs were involved in numbers of processes such as metabolic, cellular, primary metabolic, cellular metabolic and macromolecular metabolic processes (Figure 3). Some metabolic and signal transduction pathways were identified through the pathway enrichment analysis by comparing them to the whole genome background (Figure 4A). Comparing with BDs and CKs, an obvious decrease in the expression of genes was associated with photosynthesis (Table 3, Figure 4G), which is often a universal phenomenon of B deficiency treatment in plants. In addition, genes associated with the cell cycle (Table 3, Figure 4B), DNA replication (Table 3, Figure 4C), lignin biosynthesis (Table 3, Figure 4D), cytokinin signal transduction (Table 3, Figure 4E) and vascular development (Table 3, Figure 4F) were up-regulated in early or later period.

**Table 3:** Selected genes with altered expression across all libraries.

Function-al group	Database ID	Symbol	RPKM value							
			BD1	BD2	BD3	BD4	CK1	CK2	CK3	CK4
**Cell cycle**										
	orange1.1g015387m	*CYCB2;3*	1.3	3.6	14.8	6.7	4.7	1.4	1.8	3.1
	orange1.1g018657m	*CYCD1;1*	4.8	3.1	21.1	15	8	7.1	3.1	2.6
	orange1.1g021982m	*CDKB2;2*	2.8	4	17.9	8.4	3	1.5	1	3.8
	orange1.1g016078m	*CYCB1;4*	3.3	8.3	35.2	14.5	12	6.6	3.4	12.4
	orange1.1g011434m	*CYCA2;1*	1.7	1.8	9.3	2.6	0.7	1.9	1.1	1.1
	orange1.1g016163m	*CYCD3;1*	10.2	20.1	132.3	79.8	33.1	15.7	13.3	27
**DNA replication**										
	orange1.1g002353m	*MCM2*	7.4	19.1	37.1	16.4	12.6	13.3	5.5	12.4
	orange1.1g003637m	*MCM4*	3.5	7.5	15	7.5	6.1	3.6	1.8	4
	orange1.1g003809m	*MCM6*	2.8	7.3	9.4	4.1	6.9	5.4	1.6	4
	orange1.1g004502m	*MCM3*	1.6	8.5	18.1	6.8	7.6	4.6	4.5	7
	orange1.1g004862m	*MCM5*	3.2	9.1	19.2	8.5	7.5	6.4	3.1	4.7
	orange1.1g013632m	*POLD2*	13.8	18.7	24.1	29.4	16.3	15.6	11.1	16.6
	orange1.1g024527m	*PCNA2*	20.7	27.7	57	38.4	25.8	19.8	21.5	25.9
**Cytoskeleton**										
	orange1.1g013066m	*TUA2*	9.4	26	80.9	33.4	12.2	12.2	5.3	16.6
	orange1.1g013291m	*TUB8*	19.7	33.7	82.2	55.2	26.8	26.3	26.4	21.5
	orange1.1g013439m	*TUB6*	3.9	5.6	21.7	12.6	4.8	1.8	3.2	3.7
**Cytokinin signal transduction**										
	orange1.1g001846m	*WOL*	93.4	105.3	22	46.4	44.2	52.9	10.4	38.6
	orange1.1g005719m	*ARR12*	53.4	32.5	20.9	14.8	13.8	27.8	34.9	14.3
**Vascular development**										
	orange1.1g027928m	*WOX4*	67.6	57.9	304.5	218	73.4	95.1	103.7	87.2
	orange1.1g019821m	*VND7*	1.3	-	8.4	7.9	3.3	2.8	1.4	4.1
**Lignin biosynthesis**										
	orange1.1g024849m	*MYB85*	13.4	6.5	30.3	33	11.1	16.5	16.5	12.6
	orange1.1g045411m	*MYB63*	0.4	-	23.3	34.4	-	-	-	-
	orange1.1g024441m	*MYB42*	0.3	-	9.6	4.1	0.8	0.7	5.4	0.4
	orange1.1g037382m	*PAL*	5.6	4	59.7	38.7	1.4	2.9	2.8	3.8
	orange1.1g012770m	*C4H*	0.4	0.1	14.5	13.4	0.2	0.1	-	0.1
	orange1.1g042783m	*4CL2*	33.4	19.8	348.9	339.6	29	31.8	38.3	22.3
	orange1.1g026418m	*CCR*	38.9	42.9	176.1	258.9	34.8	30.9	50.4	38.2
	orange1.1g025824m	*CCoAOMT*	90.2	59.2	432.4	377	79.2	83.6	60.8	78.7
	orange1.1g020266m	*CAD*	29.3	12	83.2	69.4	21.3	14.5	15.6	36.6
	orange1.1g018871m	*PER*	10.7	10.3	388.2	186.6	15.3	12.5	4	16.9
**Photosynthesis**										
	orange1.1g017613m	*ATPC1*	632	844.5	230.9	371.9	942	873.3	952.6	1085.9
	orange1.1g019870m	*PSBO2*	74.9	121.3	38.8	58.6	134.4	108.8	156	131.9
	orange1.1g024892m	*ATPDZ*	379.2	526.9	107	180.9	718.5	522.4	364.7	651.5
	orange1.1g026780m	*PSBQ2*	62	90.6	43.4	56.9	111.7	89.2	189.6	140.1
	orange1.1g026929m	*PSAF*	8.1	8.3	5.6	8.3	11.5	15	20.7	12.9
	orange1.1g027843m	*PSAL*	1690.7	2199.2	1122.7	1613.9	2453.2	2297	5119.5	3232.8
	orange1.1g028581m	*PSAD2*	1078.4	1490.4	452.4	661.4	1570.3	1446.6	2151.9	1650
	orange1.1g029024m	*PSBY*	1155.4	1273.5	428.7	601.6	1180.8	1196.8	2557.4	1333.6
	orange1.1g030950m	*PETE1*	700.9	1207.5	475.4	793.7	1122.8	1036.6	2660.4	1525.1
	orange1.1g031508m	*PSAG*	2578.3	2862.3	1040.8	1661.6	2931.6	3269.3	4827.5	3419.4
	orange1.1g032245m	*PSAH2*	935.4	1221.4	678	737.1	1151.6	1164.6	2804.9	1688.6
	orange1.1g032691m	*PSBW*	523.1	870.4	175.4	223.1	903.9	748.1	1388.9	1196.9
	orange1.1g033067m	*PSAK*	855.8	1312	594.4	674.2	952.4	933.6	2363.3	1288.5
	orange1.1g040346m	*ATPD*	191.1	260.3	78.1	120.7	226	184.9	227.1	258
	orange1.1g043022m	*FNR2*	1112.5	2032.7	444.3	420.5	2539.4	1983.5	1883.2	2855.3

RPKM value of which gene expression was not detected in a library was indicated with -. RPKM, Reads Per kb per Million reads.

### Genes associated with major functional group were affected in corky split veins

As the global gene expression results showed that B deficiency could affect cell cycle, DNA replication, cytoskeleton, cytokinin signal transduction, vascular development, lignin biosynthesis, and photosynthesis in citrus vein (Table 3). Genes associated with cytokinin signal transduction pathway were affected in corky split veins (Figure 4E). The cytokinin receptor authentic histidine kinase gene (*HK*/*WOL*) and type-B authentic response regulator (ARR) family member *ARR12* accumulated to high levels at the BD1 and BD2 phases (Figure 4E). In addition, genes related to the three groups which were involved in cell division such as cell cycle (Figure 4B), DNA replication (Figure 4C) and cytoskeleton (data not shown in Figure 4, see Table 3) were up-regulated in the corky split veins of citrus, and these genes included *CYCs*, *CDKs*, *MCMs*, *POLD2*, *PCNA2*, and *TUs*.

Furthermore, genes involved in vascular development such as *WOX4* and *VND7* (Figure 4F) increased apparently in the corky split vein development. The dynamic accumulation of lignin biosynthesis genes (Figure 4D) during cellular differentiation also increased. These genes including *MYB85*, *MYB63*, *MYB42*, *PAL*, *C4H*, *CCR*, *CAD*, *4CL*, *CCoAOMT*, and *PER*, which encode transcription factors or enzymes involved in lignin biosynthesis pathway, were up-regulated at the BD3 and BD4 phases. Moreover, several genes, *PSAs*, *PSBs*, and *FNR2* involved in photosynthesis (Figure 4G), were remarkably down-regulated by B deficient treatment.

### Differentially expressed genes were confirmed by qRT-PCR

**Figure 5:**
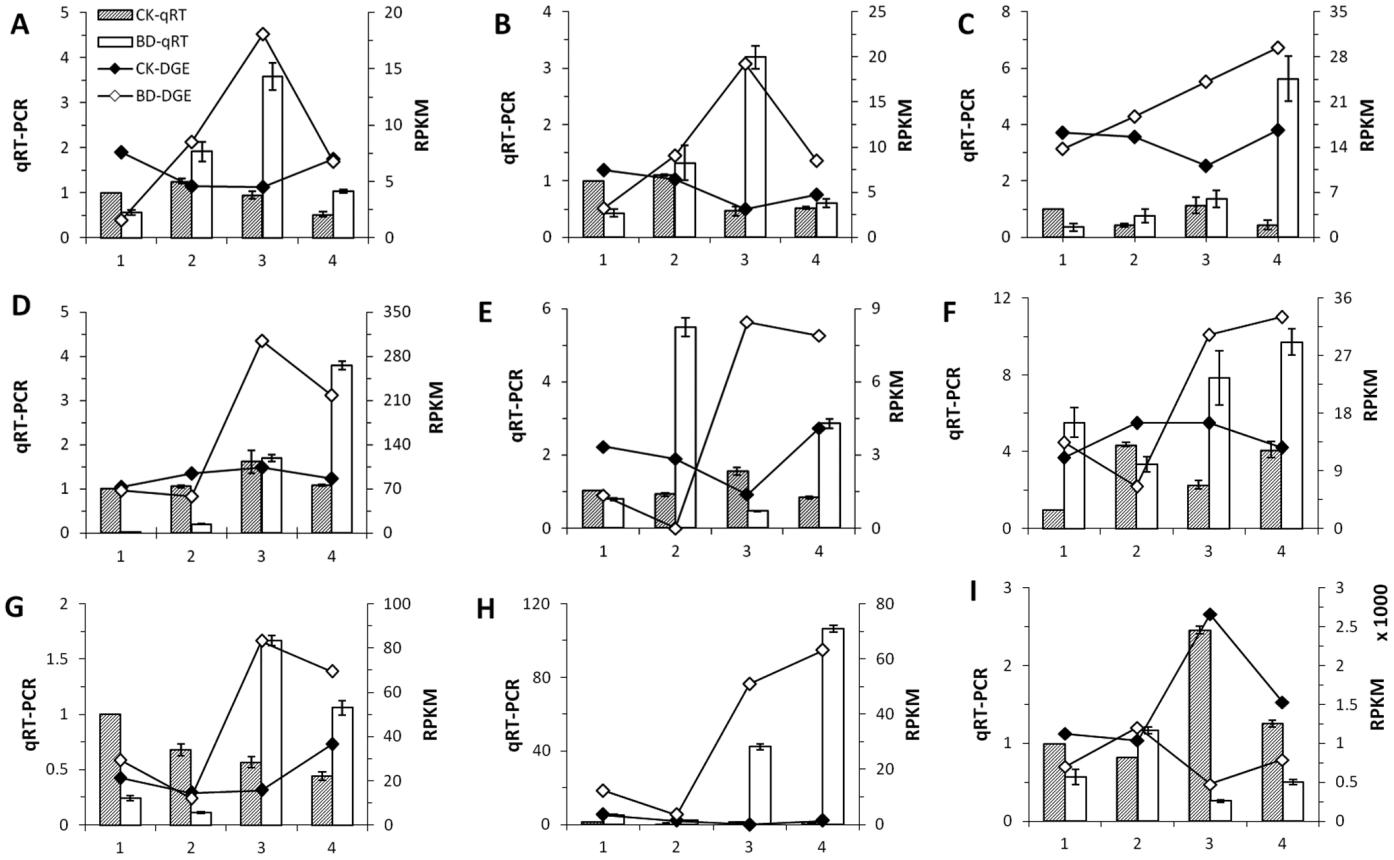
qRT-PCR confirmation of differentially expressed genes from digital gene expression analysis. [A–I] The transcript levels from qRT-PCR and digital gene expression analysis of differentially expressed genes: *MCM3* [A], *MCM5* [B], *POLD2* [C], *WOX4* [D], *VND7* [E], *MYB85* [F], *CAD* [G], *CCR* [H], and *PETE1* [I]. Relative expression levels were calculated by qRT-PCR with actin as a standard. Columns and bars represented the means and standard error of relative expression levels (n = 3) respectively (Y axis at left). Lines represented the means of RPKM value calculated by digital gene expression analysis (Y axis at right). The x-axis indicated the phases of vein development. RPKM, Reads Per kb per Million reads.

To confirm the results obtained through DGE analysis, a total of nine DEGs were selected to analyze their expression profiles by qRT-PCR in the four stages. The representative genes selected for the analysis were those involved in DNA replication, as well as vascular development, lignin biosynthesis and photosynthesis pathways. The relative expression levels of CKs and BDs were compared with those of DGE data respectively (Figure 5). Despite some quantitative differences at expression level, qRT-PCR results revealed the same expression tendency as DGE results. Eight genes (*MCM3*, *MCM5*, *POLD2*, *WOX4*, *MYB85*, *CAD*, *CCR*, and *PETE1*) expressed consistently both in qRT-PCR and DGE data (Fig. 5A–D, F–I) and only one gene (*VND7*) by using qRT-PCR did not agree well with the DGE analysis pattern (Fig. 5E).

## Discussion

In this study a symptom of corky split vein was observed in B-deficient citrus. In addition, the surveys on morphology, anatomy, and transcriptome were performed at the four stages of corky split veins to compare to the control. The histological micrographs indicated that vascular hypertrophy was related to the symptom occurrence. Through the use of high-throughput sequencing, we mapped in details the transcriptional changes that occurred during hypertrophic development. Genes associated with plant cytokinin signal transduction, cell division, vascular development were affected in corky split veins.

### Corky split vein caused by B deficiency was the result of vascular hypertrophy

The deficiency of B in citrus leaf was defined on the condition that B concentration was no more than 20 mg kg^−1^DW [47]. B concentrations in mature leaves were determined (Figure 2). In the views of previous research, the situation of 8.86 mg kg^−1^DW measured in BD leaf belonged to the level of B deficiency. Meanwhile, the symptom of corky split vein (Figure 1D, E) was similar to vein of B-deficient mulberry leaves [9]. Based on their morphologic characters, the four stages were selected for the following analysis and named BD1, BD2, BD3 and BD4, respectively. Vein in the BD1 stage (Figure 1B) was similar with CK veins (Figure 1A). Vein at the BD2 phase (Figure 1C) slightly protruded from the blade compared with CK vein (Figure 1A). Corking vein was clearly observed in the BD3 stage and it protruded from the blade prominently (Figure 1D). In the BD4 stage, the leaf vein exhibited seriously corky split (Figure 1E). In addition, micrographs of light microscopy on LVs were observed through the four stages (Figure 1F–J). Compared with CK (Figure 1F), the area of vascular tissues at four phases (Figure 1G–J) became larger with treating time extending. Interestingly, due to the vascular hypertrophy, epidermis was broken at the BD3 phase and then expanded extremely at the BD4 phase. Thus cracked vein could be found through morphological detection when the epidermis was destroyed. Therefore, these dynamic observations suggested that corky split vein caused by B deficiency was the result of vascular hypertrophy.

### Global gene expression was changed during the corky split vein development

DGE profiling could facilitate the identification of systemic gene expression. It reveals quantitative changes in transcript abundance on a genome-wide scale. Here, 8 transcriptomic profiles of vein were performed to identify differentially expressed genes in the four stages of corky split vein. A sequencing depth of more than 7.1 million reads per library (Table 1) was reached, and highly similar tendencies of randomness assessments (Figure S3) were analyzed in eight libraries, suggesting that the sequencing experiment was successful. To our konwledge, it is the first time that changes in transcriptomic profiles are shown in corky split vein when the naval oranges were subjected to long-term B deficiency.

Results of DGE analyses showed that we could effectively identify DEGs across a wide range of transcript abundances. The global gene transcription in the stressed leaf vein significantly altered 418–3050 genes assayed during the four developmental stages of corky split vein (Table 2). Many of the genes are known to be responding stress and stimuli (Figure 3). Decreased expression of photosynthesis was observed in this study (Table 3, Figure 4G), which is similar to the results of previous physiological experiments on other B-deficient plants [48], [49].

### Cell division and vascular development were involved in the formation of corky split vein

Vascular hypertrophy caused the formation of the corky split vein by the intensifying of cell division and cell differentiation. Numerous studies on plants showed that the plant cell cycle is governed by cyclin-dependent kinases (CDKs) that are associated with their activator proteins called cyclins (CYCs), and the activity of CYC–CDK is modulated at both transcriptional and post-translational levels at the G_1_-to-S and G_2_-to-M transitions [50], [51]. *CDKB2*, one of the plant-specific B-type CDKs (CDKBs) genes, its expression was specific to the G_2_ and M phases [52], [53]. A recent study demonstrated that *CDKB2;2* was required both for normal cell cycle progression and for meristem organization in *Arabidopsis thaliana* [54]. Genes involved in cell cycle in our experiment were differentially expressed (Table 3). The expressions of cell cycle genes were up-regulated at the BD2, BD3 and BD4 stages (Figure 4B). Thus up-regulated expressions of cell cycle genes may contribute to the proliferating vascular cylinder.

*WOX4*, a member of the *WUSCHEL-related HOMEOBOX* (*WOX*) gene family, is required for promoting the proliferation of procambial/cambial stem cells in *A. thaliana*. Compared with Col-0, the mutant *wox4-1* displays less procambial cell number and smaller stele width [55]. The function of *WOX4* on maintaining procambial/cambial stem cell activity has been also confirmed in *Vitis vinifera* L. [56] and *Solanum lycopersicum* [57]. *VND7*, a member of *Vascular-Related NAC Domain* gene family, acts as a positive regulator for protoxylem vessel formation in *A. thaliana* [58], [59]. In vascular vessels, *VND7* controlls both secondary wall development and programmed cell death (PCD) of the vessels in both root and shoot tissues [60], [61]. Over-expression of *VND7* could induce transdifferentiation of various cells into protoxylem-like vessel elements in both *A. thaliana* and *Populus tomentosa*. In addition, PCD-related gene transcripts are highly accumulated in *VND7* over-expressing plants [59]. In this study, expressions of *WOX4* and *VND7*, were highly increased along the development of corky split vein (Figure 4F), implying that the formation of corky split vein under B-deficient condition was related to the expression alteration of the two vascular development genes (Figure 4A).

### Cytokinin performs a role in initiating the vascular hypertrophy

In previous physiological experiments, the plant phytohormone cytokinin promotes the differentiation of tracheary elements [62], [63] and contributes to the maintenance and proliferation of cambial cells [64], [65]. It is generally believed that cytokinin executes these physiological activities by regulating cell division and cell differentiation through cytokinin signal transduction pathway [66], [67], [68], [69]. In *A. thaliana*, WOL, one of primary cytokinin receptors, is necessary for early procambial cell division in embryogenesis [70]. The *wol* mutant exhibits a reduced cell number and a cell division process failing to take place in the root and lower hypocotyl region soon after the torpedo stage [71]. Type-B ARRs are implicated in the cytokinin signal transduction pathway and acts downstream of the cytokinin receptors [66], [67], [68], [69]. As transcriptional activators, the phosphorylated type-B ARRs could induce rapid transcription of cytokinin-associated target genes [72]. Elegant genetic studies in *A. thaliana* showed that ARR12, one of type-B ARRs, redundantly played an important (or essential) role in cytokinin signal transduction [73], [74]. An *arr10 arr12* double mutant exhibited the inhibition of root elongation [73] and an *arr1 arr10 arr12* triple mutant displayed stunted growth and abnormality in vascular development [74]. Our results showed that the two genes, *WOL* and *ARR12*, were up-regulated at the BD1 phase in corky split veins (Figure 4E) and these indicated that the cytokinin signal transduction pathway may play a role in initiating the vascular hypertrophy/observed phenotype.

## Conclusions

In summary, our research described changes in the transcriptome of leaf veins during four stages of exposure to B deficiency using DGE analysis. In this study a symptom of corky split vein was observed in B-deficient naval orange. The histological micrographs indicated that vascular hypertrophy was related to the symptom occurrence. Through the use of high-throughput sequencing, we mapped in detail the transcriptional changes that occurred during hypertrophic development. The changes in the pathways were usually consistent with the observed symptoms. Genes associated with plant cytokinin signal transduction, cell division, vascular development were affected in corky split veins. The cytokinin signal transduction pathway may play a role in initiating the observed phenotype. This research provides a new insights into the molecular mechanisms of corky split vein caused by B deficiency.

## Supporting Information

Figure S1 **Examples of collecting samples for total RNA extraction (whole) and light microscope observation of vein (asterisked in white).** Whole leaf vein was used for total RNA extraction experiment. The position of lateral vein, at the 3^rd^ item near the petiole, for light microscope observation was marked using a white asterisk.
(TIF)

Figure S2 **Classification of raw reads of eight libraries.**
(TIF)

Figure S3 **Randomness assessments of the eight libraries.** [A-H] Randomness assessments of library CK1 [A], BD1 [B], CK2 [C], BD2 [D], CK3 [E], BD3 [F], CK4 [G] and BD4 [H].
(TIF)

Figure S4 **Sequencing saturation analysis of the eight libraries.** [A–H] Sequencing saturation analysis of library CK1 [A], BD1 [B], CK2 [C], BD2 [D], CK3 [E], BD3 [F], CK4 [G] and BD4 [H]. With the number of reads increasing, the number of detected genes was increasing in the eight libraries. However, when the number of reads reached 3 million reads or higher, the growth rate of detected genes became flatten.
(TIF)

Table S1 **Primer sequences for qRT-PCR.**
(XLSX)
