# Supplementary figures and images for: Digital Gene Expression Analysis of Corky Split Vein Caused by Boron Deficiency in ‘Newhall’ Navel Orange (Citrus sinensis Osbeck) for Selecting Differentially Expressed Genes Related to Vascular Hypertrophy
Source: PLoS One. 2013 Jun 5;8(6):e65737. doi: 10.1371/journal.pone.0065737 (PMC3673917; doi:10.1371/journal.pone.0065737)

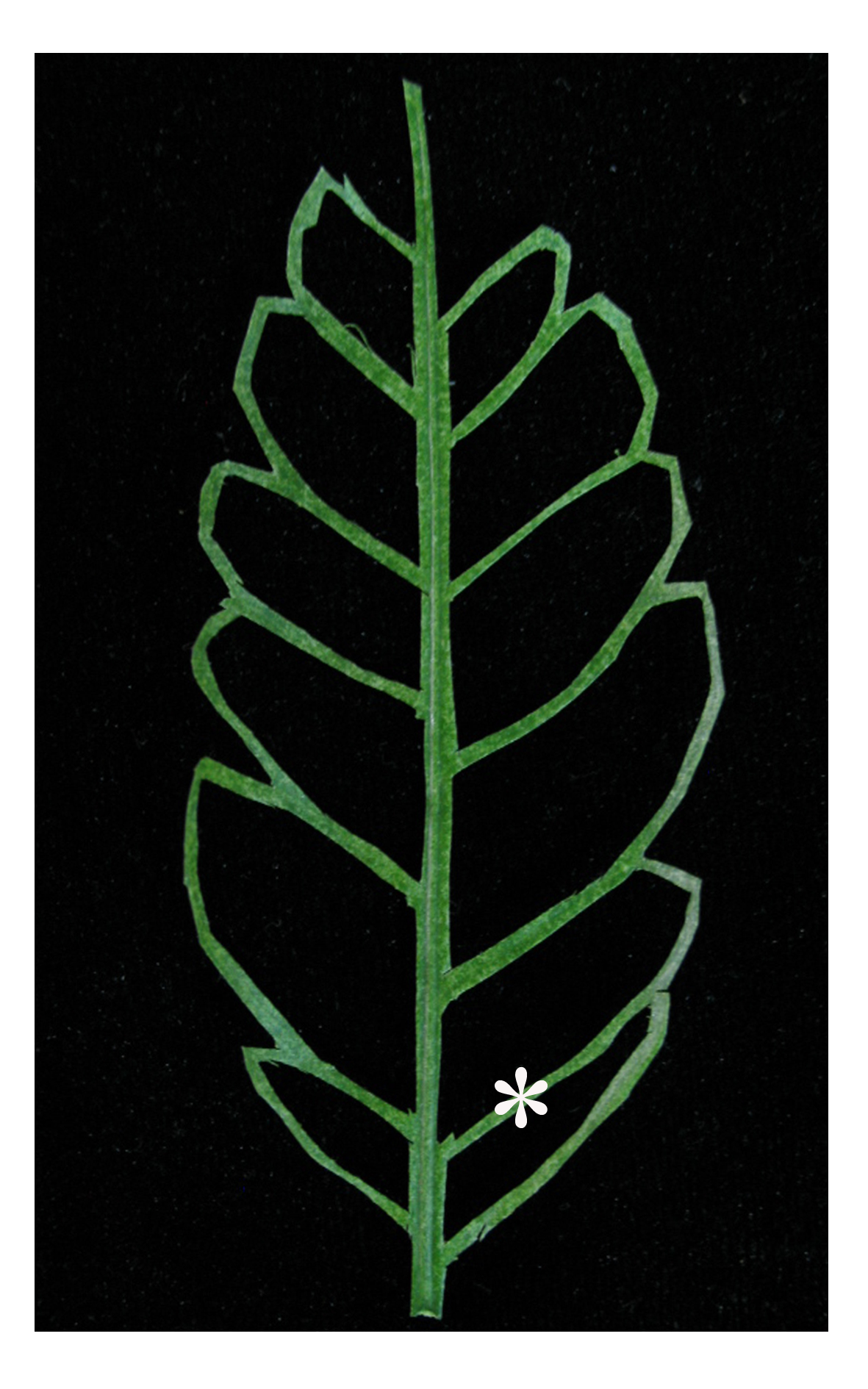

Supplement: Figure S1 — Examples of collecting samples for total RNA extraction (whole) and light microscope observation of vein (asterisked in white). Whole leaf vein was used for total RNA extraction experiment. The position of lateral vein, at the 3rd item near the petiole, for light microscope observation was marked using a white asterisk. (TIF) [file pone.0065737.s001.tif]

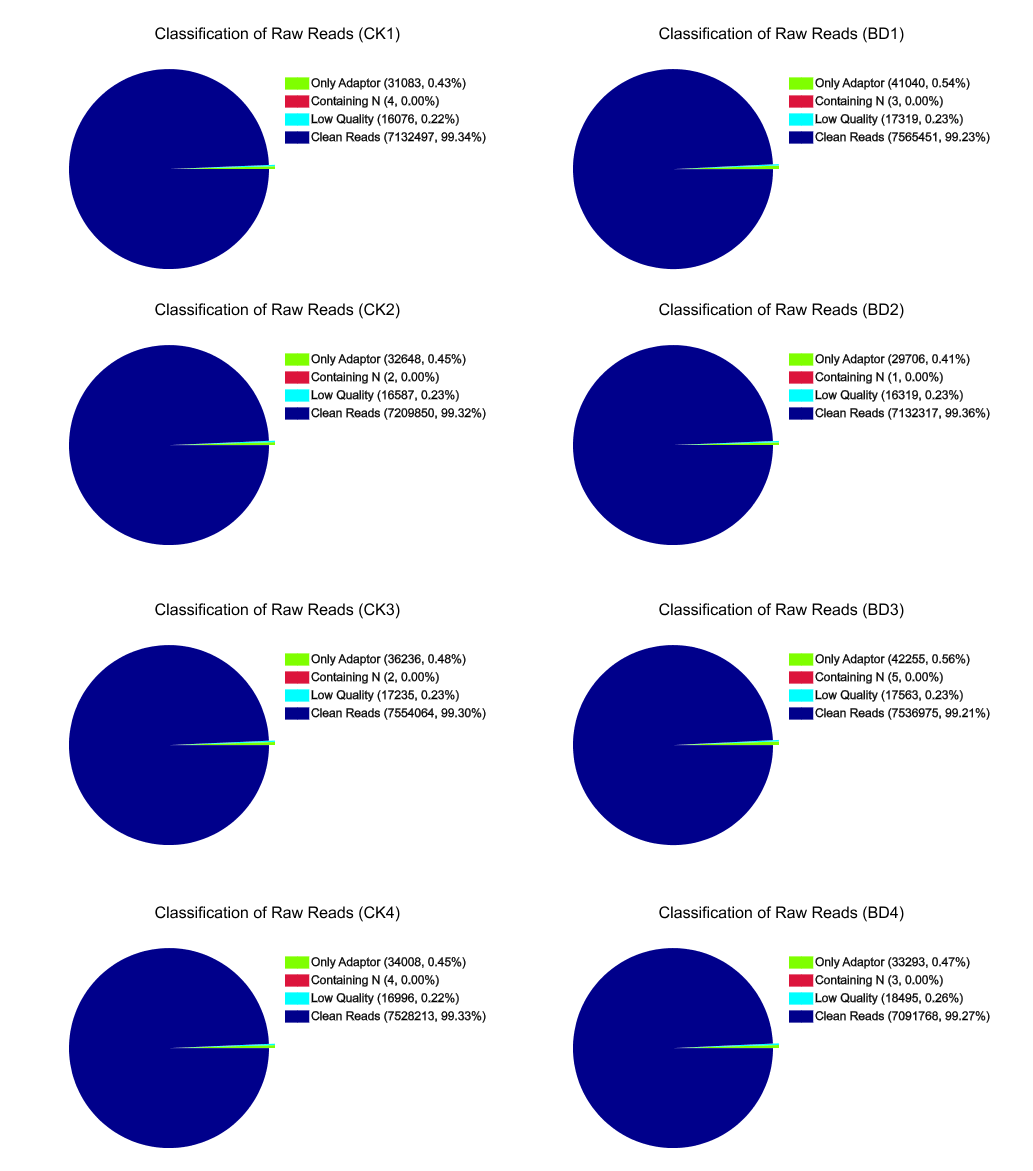

Supplement: Figure S2 — Classification of raw reads of eight libraries. (TIF) [file pone.0065737.s002.tif]

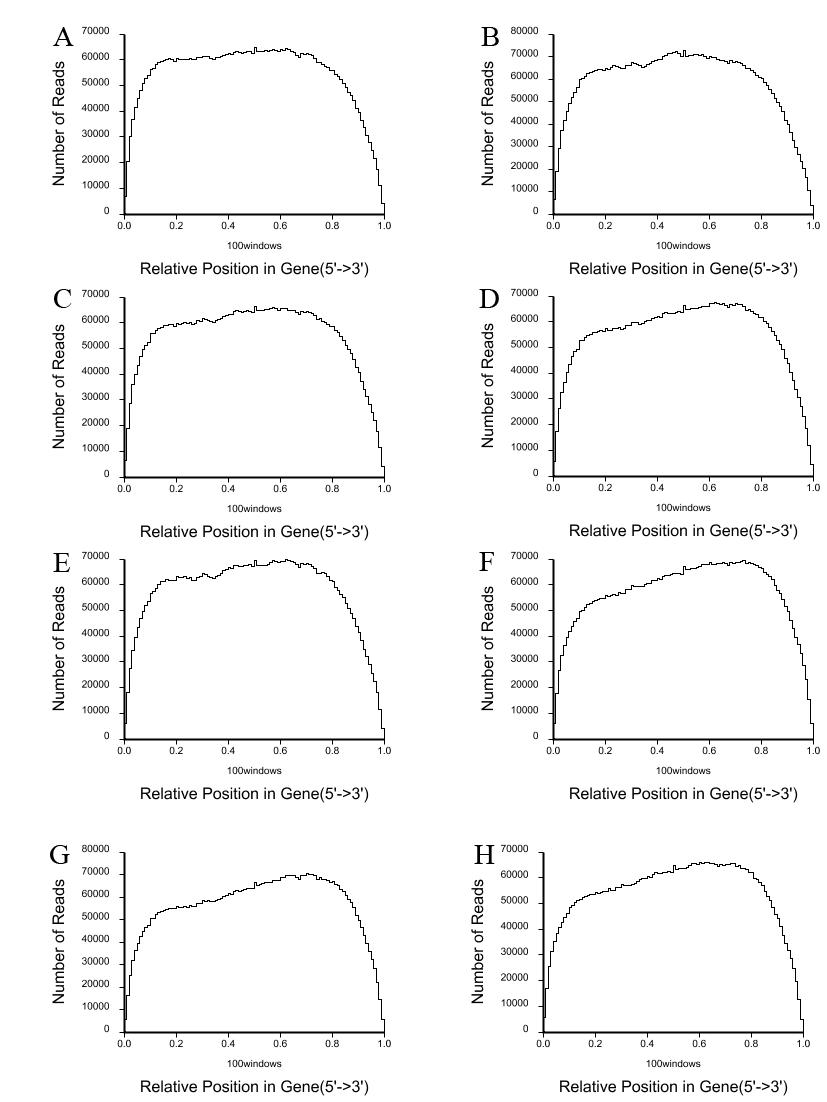

Supplement: Figure S3 — Randomness assessments of the eight libraries. [A-H] Randomness assessments of library CK1 [A], BD1 [B], CK2 [C], BD2 [D], CK3 [E], BD3 [F], CK4 [G] and BD4 [H]. (TIF) [file pone.0065737.s003.tif]

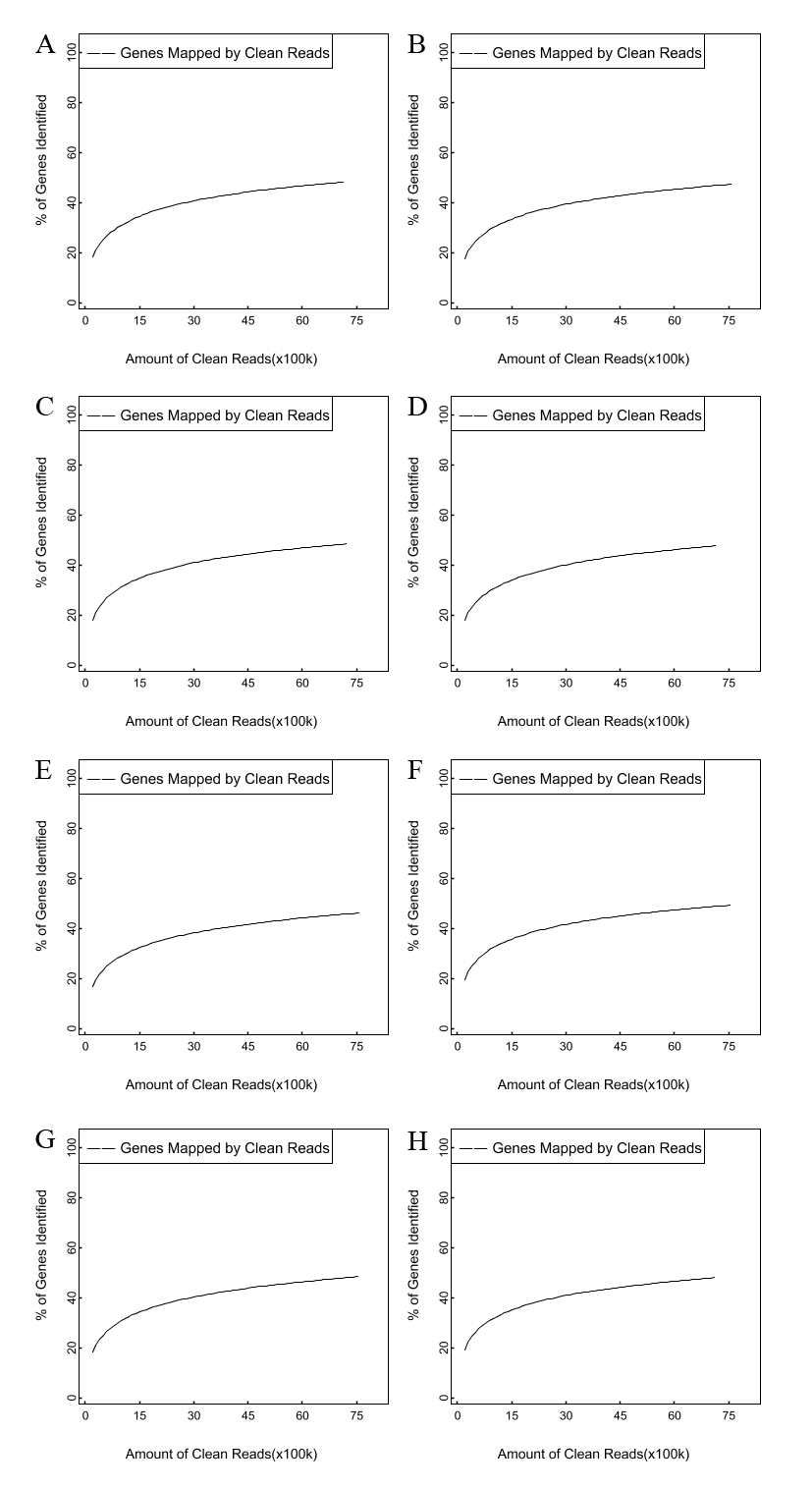

Supplement: Figure S4 — Sequencing saturation analysis of the eight libraries. [A–H] Sequencing saturation analysis of library CK1 [A], BD1 [B], CK2 [C], BD2 [D], CK3 [E], BD3 [F], CK4 [G] and BD4 [H]. With the number of reads increasing, the number of detected genes was increasing in the eight libraries. However, when the number of reads reached 3 million reads or higher, the growth rate of detected genes became flatten. (TIF) [file pone.0065737.s004.tif]
